# Arteriosclerosis—Etiology, Diagnosis and Treatment

**Published:** 1911-05

**Authors:** J. H. Neall

**Affiliations:** Atlanta, Ga.


### ARTERIOSCLEROSIS—ETIOLOY, DIAGNOSIS AND TREATMENT.

By J. H. Neall, M. D., Atlanta, Ga

Arteriosclerosis or endarteritis deformans is a chronic degeneration and inflammatory disease of the arterial system.

A certain amount of hardening takes place normally in the human subject as advancing age comes to the individual. Arteries that would be considered normal for a child would not be so for the youth and those of the youth would not be normal for the old man. Hence the statement that a man is as old as his arteries, holds good in almost every case. Some, as Dr. Osler puts it, may have poor material used in the body tubes in the first place and consequently offer an exception to the rule.

Pathologically, then, arteriosclerosis is a condition of thickening, diffuse or circumscribed, beginning in the intima, but which later involves the other coats. The process leads, in the large arteries, to what is known as atheroma and to endarteritis deformans.

### Etiology.

By Virchow the disease was supposed to be due to a true inflammatory hyperplaisa as the result of some "formative stimulus;" and by Thomas to be a compensatory thickening of the walls in order to diminish the lumen of the vessel after the stretching which occurred under the increased blood pressure with which it was usually associated.

Dr. Osler mentions five things that tend to the production of this condition in those endowed with the usual amount of good material in their make-up: (1.) Chronic Intoxications, alcohol, lead, gout, and syphilis. (2.) Overeating. (3.) Overwork of the muscles. (4.) Renal Disease. (5.) As an involution process, an accompaniment of old age.

Rogers says: I think it is a well-established fact that gastro-intestinal disturbances, when characterized by fermentation and putrefaction, induce arterial changes. In other words, one potent factor in the causation of this disease is autointoxication. The liver is the organ generally most profoundly affected by the poisons thus generated; sooner or later these poisons pass the barrier opposed by this gland and exert their action upon other parts of the organism, notably upon the arteries and kidneys.

A most interesting sidelight upon the origin of arteriosclerosis has been thrown by attempts to produce it experimentally in animals, especially in rabbits and guinea pigs. Hirchfelder says that the lesions which have been produced cannot be termed true arteriosclerosis like that seen in man, but are confined to the media and aventitia, the intima always remaining clear The reason for this is not evident.

Gilbert and Lion have been able to produce arteriosclerosis in animals by the injection of bacterial toxins, and this has been confirmed by Klotz. The demonstration of this fact is of great importance not only from the standpoint of experimental arteriosclerosis, but also because it establishes the importance of bacterial disease in the etiology of arteriosclerosis met with clinically.

The earliest observations of arteriosclerosis brought about by toxic action of organic compounds, and one which establishes beyond doubt the deleterious action of tobacco upon the arteries, is that of Isaac Alder, demonstrating sclerosis in the smaller peripheral arteries of rabbits by giving infusion of tobacco by the stomach. Boverie confirmed these results by giving infusion of tobacco by stomach-tube, and obtaining artheromatous plaques or thickening at the base of the aorta in ten out of sixteen rabbits, while Baylac obtained sclerosis in each of eight rabbits into which tobacco infusion was injected intravenously or subcutaneously.

Jebrousky, and later, W. E. Lee, have produced it in rabbits made to inhale tobacco smoke. From these experiments it would seem that the channel by which the poison enters the body bears some relation to the location of the lesion and to the lesion itself.

This may explain the very marked action of tobacco inhaled and entering the heart directly from the pulmonary circulation in smokers as compared with the somewhat milder effects of chewing tobacco, under which condition the nicotine passes through and is perhaps somewhat attenuated in the liver before entering the systemic circulation, and has still to pass through the venae cavae, right heart and pulmonary circulation before reaching the coronary artery.

"In smoking, however, the nicotine enters through the lungs and strikes its first blow at the coronary arteries and base of the aorta where the elastic fibres are under the greatest strain and hence most liable to degeneration. It is, therefore, easy to understand why smoking of heavy cigars should be one of the most potent factors in the etiology of arteriosclerosis and coronary chlerosis."

Let us reert to the bacterial causation of arteriosclerosis from bacterial toxins. It is a well-known fact that the faeces of animals that are carnivorous, have in them many millions more of bacteria than the faeces of animals that are vegetarians or frugivorous. This is shown not only by the miscroscope, but is demonstrated by the much stronger mal-odors from urine and faeces of carnivora than from the others above mentioned. Take, for instance, the cat, lion, tiger, in contrast to the horse, elephant, sheep and bovine speçies.

We know that ptomaine poison can come only from the ingestion of putrescent meat and we know that meat remains long enough in the alimentary canal to undergo a degeneration closely bordering on that dangerous state. The only thing that saves the carnivorous animals is the fact that they secrete a sufficiently antiseptic digestive fluid to destroy enough bacteria to bring it within safe limits. They have also a much shorter alimentary canal.

Our ancestors were in much less danger than we, not only from the standpoint of more disease in the animals at present, but also that their vigorous outdoor life enabled them to manufacture a much stronger antiseptic digestive fluid than is possible for us at this day and age.

It is a question whether continued high blood pressure produced by vigorous exercise alone would ever cause arteriosclerosis but high blood pressure with tobacco, alcohol, and autointoxication from wrong habits of eating, together with hard work, will almost certainly, sooner or later, show this lesion.

The gluttinous high living, with the strenuous life in the United States, is making itself known in the increased cases of arteriosclerosis and apoplexy.

Buttner, quoting from Albutt, with whom he is in accord, says that "one main cause of rising arterial pressure in middle life is excess of feeding, that is to say, of food in excess of work and excretion." * * * "For the statement that meat-eaters are more prone to arteriosclerosis, we have no positive warrant, but the Indians and Japanese, who subsist chiefly on vegetable diet, are said to be much less affected than Europeans.

Houchard, in his Clinical Treatise of the Maladies of the Heart and of the Aorta, "is more positive with regard to the influence of meat and is convinced of its harmful effects."

Speaking of this conviction, he says: "A very frequent cause is the diet * * * I was writing already in 1889. I am convinced that excesses and errors of diet, by introducing in the body many toxic substances, such at ptomaines, are a frequent cause of arteriosclerosis, especially when those substances are not quickly excreted by the kidneys, which had become inefficient. In a word, a number of bodies found in food have toxic properties, some act more strongly on the voluntary muscles, some, more on the blood-vessel muscles. The result is that the arteries are in a state of more or less permanent spasm, which produces hypertension and arteriosclerosis. The therapeutic conclusion is that we must prescribe a regime from which are excluded foods more or less contaminated with ptomaines and excretive matter." * * * Two years later (1899) Dujardin Beaumetz exposed the same ideas, for he said in speaking of the toxic origin of this affection that "these substances (ptomaines and leucomaines) produced by the body, irritate the arterial walls and gradually modify them." "We have nothing to add to this quotation,' said the latter, to which we agree, except that ptomaines are not only produced by our body, but we introduce them in large quantities into our organism by our food." * * * From the idea that renal insufficiency is an early and almost unfailing symptom of arterial cardiopathies, I thought that they might also be a cause. Indeed, the kidney has not an unlimited capacity of elimination. When too much poison is introduced in the organism, a certain amount can not be completely eliminated, the kidney is incapable of carrying on the excessive work imposed upon it, thus is constituted a relative insufficiency. It is relative because it is not dependent on the organ which is still in perfect condition, but of the amount to excrete. The disease begins in an intoxication, continues and ends in an intoxication.

Any substances entering the circulation that irritates the membranes of the arteries, as delicate and sensitive as the conjunctiva of the eye, will set up an inflammation and thereby weaken the arterial wall. This may go on to the attempt of nature to repair the damage by depositing lime in the wall to strengthen it.

Among these irritating substances we may classify tobacco, alcohol, mustard, pepper sauce, ginger, capsicum, cayenne, horse-radish, and all the spices, condiments and things of that sort—tea and coffee are all causes for the hardening of the arteries.

Professor Huchard of Paris, who is the world's greatest authority on the question of blood-pressure and arteriosclerosis, as well as German investigators, has made an experimental study of the subject. They took the various kinds of spices and condiments and made extracts from them and injected these extracts into the veins of rabbits, dogs, and other animals and at the end of four months they found patches of lime deposits in the walls of the arteries.

A cup of coffee contains much more of caffein than the same amount of urine contains of uric acid and the caffein and uric acid are practically equivalent. Uric acid has oxyzanthin and caffein that coffee contains, is trimethilzanthin; they are both zanthins and are practically the same thing.

Blood pressure is never higher than is necessary to overcome the resistance in the arteries and the capillaries. The use of tobacco and the eating of meats causes a contraction of the peripheral blood vessels. Alcohol and the nitrates dilate them, hence the relief experienced by the smoker taking his whiskey, beer or wine. Who does not well remember the deathly paleness that was experienced at his first attempt in using the filthy weed. Here was tobacco getting in its deadly work in paralyzing the vaso-motor nerves, causing the capillaries to contract and shut off the circulation at the surfaces at least. Nature acquires a toleration, it is true, but the deadly work goes on.

### Diagnosis.

The feeling of superficial arteries may give a clue to the condition. The sclerosed artery is rigid, non-contractile and inelastic and the part it supplies cold, congested, ill-nourished and often edematous.

Very often in this condition high blood pressure may be detected. The sphygmomanomiter is absolutely essential to clearing up this important point, and it should be invariably employed as a means of diagnosis in every case of arterial disease. It is practically impossible that the unaided finger, no matter how skilled the observer may be in pulse reading, to determine with accuracy how much of the hardness and firmness of an artery is produced by high blood pressure and how much is due to the thickening of the arterial wall.

The ordinary clinical type of arteriosclerosis is not necessarily accompanied by high blood pressure, a large percentage failing to show this development.

On physical examination the radial arteries may or may not be found to be thickened or beaded (atheromatous), dependent partly upon the distribution of the sclerosis, since the radial artery may be spared.

Some writers state, however, that in men who do hard manual labor the radial arteries are the first attacked, while in those who lead a sedentary life sclerosis may appear very early about the base of the aorta, and the radial, nevertheless, be perfectly normal.

Changes in the retinal vessels constitute an early sign of arteriosclerosis, as pointed out by Hirschberg in 1882, who later demonstrated this change was normal in old persons and usually began in the fifth decade.

X-ray gives absolute proof of arterioscierosis by which calcified plaques along the course of the deeply situated arteries may be discerned as distinct shadows along the course of the arteries. It has not been possible to discern sclerosis in the coronary arteries in this way.

There may be pain over the precordium, in the shoulders or down the arms, or in the abdomen or legs, which may be associated with periods of high blood pressure.

It is quite impossible in the short time that is granted to a paper to go exhaustively into the various phemonena associated with this condition.

### Treatment.

The following is an abstract from an article written by Houchard, entitled, "Prophylactic Treatment of Arteriosclerosis," given in the "Bulletin de l Academie de Medicine," Paris:

Huchard has been asserting that certain functional disturbances of the blood vessels, the result of some intoxication, if not combatted in time lead to the anatomic changes and symptoms of arteriosclerosis.

He calls this state of arterial hypertension, the stage and presclerosis, and insists that appropriate treatment may cure it and prevent the impending arteriosclerosis. He is unable to present pathological anatomic data to sustain this view, but from the standpoint of therapeutics and the success of appropriate treatment, he claims that these assertions have been amply confirmed. The physician's task is to recognize the "condition" early, to determine the degree and the greater or less permanency of the arterial hypertension, and of the accompanying, more or less pronounced, insufficiency on the part of the kidneys.

He should strive to remove the cause, which is generally poison, from the digestive tract, acting on the coats of the vessels and irritating the kidneys. He should restrict the patient to a milk and vegetable diet, limiting the intake of salt, reducing to the minimum the alimentary toxins in the organism and promoting their elimination early and always by suitable treatment of the kidneys and diuresis, and finally, by sustaining the heart in its incessant struggle against the peripheral obstacles. Treatment along these lines, he declares, will ward off the anatomic lesions otherwise inevitable from the almost uninterrupted overwork on the part of the vessels and the numerous complications which are the result of intoxication on the organism.

Huchard dwells more particularly on the necessity for assisting the kidneys and the heart.

We see, then, that the treatment of arteriosclerosis is mainly prophylactic, hygienic, and dietetic, and actual specific treatment is of the least importance.

Says Hirschfelder: Carefully selected diet is a most important factor, with restriction in both quality and quantity. He also says that the diet should be "near the lower level for proteids, and as free as possible of purin bodies (nitrogenous extractives, such as are found in meat), creatinin, etc., and also salt. He seems to think that salt mackerel of Boston is as dangerous as the beer of Milwaukee. For the schlerotic danger probably lurks in the Smithfield ham or cold smoked tongue as well as the Baltimore rye or the Martini cocktail (Beyer, Barie, Hadfield).

Tobacco, alcohol, tea, and coffee should be dispensed with. The liquid intake should not be excessive.

### Hydrotherapy.

Systemic hydrotherapy is of considerable value in arteriosclerosis, especially the use of warm baths, alternating hot and cold douches, percussion douches, applied both locally and generally.

In most cases the effect of a good warm douche or warm bath, is more marked and more lasting than that of any of the nitrites usually made use of for the purpose of dilating the capillaries and lowering the blood pressure.

Warm baths, and alternating warm and cold showers, the so-called Scotch douche, once or twice a day are an indispensable part of the treatment of every arteriosclerotic. Venesection is not only useless but harmful.

